# Exploring the Role of Cognitive Reserve and Human–Animal Interaction in Late-Life Depression: A Moderation Analysis

**DOI:** 10.3390/geriatrics10040089

**Published:** 2025-07-01

**Authors:** Nathália Saraiva de Albuquerque, Natália Silva Sessegolo, Carmen Moret-Tatay, Tatiana Quarti Irigaray

**Affiliations:** 1 Doctoral School, Catholic University of Valencia San Vicente Mártir, San Agustín 3, Esc. A, Entresuelo 1, 46002 Valencia, Spain; 2 Department of Psychology, Pontifical Catholic University of Rio Grande do Sul, Ipiranga Avenue, 668 Partenon, Porto Alegre 90619-900, RS, Brazil; natalia.sessegolo@edu.pucrs.br (N.S.S.); tatiana.irigaray@pucrs.br (T.Q.I.)

**Keywords:** human–animal interaction, cognitive reserve, depressive symptoms

## Abstract

**Background/Objectives:** Depression impairs the quality of life in older adults and represents a significant public health issue. Cognitive reserve may act as a protective factor against depressive symptoms in older adults. Additionally, interaction with pets may serve as another potential protective factor against these symptoms. Thus, this study aimed to evaluate whether higher cognitive reserve could predict a reduction in depressive symptoms in older adults and to investigate the moderating role of pet companionship in this relationship. **Methods:** The following instruments were used: a Sociodemographic Data Sheet, the Modified Telephone Interview for Cognitive Status (TICS-M), the Cognitive Reserve Index Questionnaire (CRIq), and the Geriatric Depression Scale (GDS-15). Data were collected via video calls through WhatsApp and analyzed using a moderation analysis with PROCESS for SPSS. **Results:** The final sample consisted of 215 older adults with a mean age of 69.13 years (SD = 6.89). Among the participants, 53% owned pets and 47% did not. The overall model revealed a significant association between the predictor variables and the outcome (F(3, 211) = 4.24, *p* < 0.01). For the group without pets, the effect was not significant (β = −0.1082, *p* = 0.2916), but for the group with pets, the effect was substantial and negative on the GDS (β = −0.1936, *p* < 0.05). **Conclusions:** We concluded that the relationship between cognitive reserve and depressive symptoms is moderated by the presence of pets in individuals’ lives. These findings highlight the role of pets in protecting against depressive symptoms in older adults. Future studies should explore this relationship with more diverse samples.

## 1. Introduction

Depression in older adults poses a significant public health concern, with substantial impacts on individuals’ quality of life and societal burden [1,2]. While the etiology of late-life depression is multifaceted, recent research has underscored the potential role of cognitive reserve—a concept encompassing the brain’s ability to optimize performance through neural plasticity and efficiency—in mitigating depressive symptoms [3,4].

Late-life depression is an increasingly prevalent public health concern, with rates that have risen over recent years. Although the prevalence among older adults varies widely across different studies [5], a recent review estimated the global prevalence of depressive symptoms in older adults at 28.4% [6]. This percentage can vary significantly across continents, potentially influenced by the economic and cultural development of each region. In Brazil, a survey conducted by the Brazilian Institute of Geography and Statistics found that the age group most proportionally affected by depression was older adults between 60 and 64 years, with 13.2% having received a diagnosis [7].

The effects of late-life depression extend far beyond emotional distress, with significant societal and individual consequences. It is associated with increased use of health services and associated costs [2]. Additionally, depression in older adults often deteriorates physical health, contributing to the onset or worsening of chronic diseases and functional limitations, which impair the quality of life in this population [1,8]. Depressive symptoms are also correlated with an increased risk of executive function decline, which can negatively affect the cognitive abilities of older adults [9].

One way to mitigate the consequences of depression in older adults is through identifying protective factors against this mental disorder. A systematic review indicated that engaging in physical activities is associated with a lower risk of depression in older adults [10]. Another study observed that engaging in light physical exercises, attending classes, using a computer, and participating in community events have protective effects against depressive symptoms in older adults [11]. Furthermore, factors such as cognitive activity, social support, and a sense of purpose can serve as preventive strategies against depression in older adults [10].

Cognitive reserve has also been identified as a protective factor against depressive symptoms. It is a theoretical concept that describes the set of cognitive resources accumulated by an individual throughout their life, allowing for a delay in the effects of healthy aging and slowing the progression of neurodegenerative diseases [12]. According to Stern, cognitive reserve is divided into two models: the passive model, which pertains to structural brain characteristics and the capacity to tolerate neuropathological damage before clinical manifestations arise [13,14], and the active model, which encompasses life experiences that promote cognitive engagement and facilitate the formation and reinforcement of neural networks [15]. From a physiological perspective, cognitive reserve is supported by neurobiological mechanisms such as neurogenesis, angiogenesis, resistance to apoptosis, and enhanced synaptic plasticity. These processes strengthen the brain’s ability to reorganize and compensate for damage, thereby preserving cognitive functioning in the face of aging and pathological alterations [16].

According to a recent study, participants with higher cognitive reserve showed a reduced risk of developing depressive symptoms over time, suggesting that higher cognitive reserve may provide a protective mechanism against depression in older adults [4]. Another study revealed that cognitive reserve may mitigate cognitive impairments in individuals with major depression, with neuroprotective effects of cognitive reserve observed in functions such as working memory, inhibitory control, cognitive flexibility, and attention [3].

Another potential protective factor against depressive symptoms is interaction with pets. Although research results are still inconsistent, an increasing number of studies highlight the positive role of animals in mental health. Individuals who own pets have shown lower stress levels and higher positive mood levels after interacting with their animals [17]. Moreover, pet companionship is associated with greater social participation [18]. Research also shows that pet caregivers experience fewer depressive symptoms compared to those without pets [19,20]. This correlation has also been identified in studies involving older adults. For instance, one study observed that among older adults who had faced a divorce or the death of a spouse, pet companionship was related to reduced depressive symptoms, and during the COVID-19 pandemic lockdown, another study showed that older adults with dogs exhibited lower levels of depressive symptoms [21,22]. However, it is important to note that some studies have found no significant mental health benefits or have even suggested that individuals with pets may report higher levels of psychological distress [23,24]. These mixed findings underscore the complexity of the relationship and highlight the need to consider issues of causality and potential confounding factors.

The exact mechanism by which pets contribute to their caregiver’s mental health is still unknown. One hypothesis involves the role of oxytocin in these interactions. Oxytocin is a neuropeptide produced in the hypothalamus and is important for attachment between mothers and offspring in mammals, facilitating bond formation [25]. Evidence indicates that interactions with dogs increase oxytocin levels in humans [25,26]. Notably, Nagasawa et al. (2015) demonstrated that mutual gaze between dogs and their caregivers increases oxytocin levels in both species, suggesting the presence of a bidirectional, oxytocin-mediated bonding mechanism [27]. The reduction in fear and anxiety, along with decreased physiological stress parameters such as heart rate and blood pressure, is associated with the activation of the oxytocin system. This process may be the mechanism responsible for many of the positive effects of human–animal interactions, promoting psychological and physical well-being [28].

This research explores the nuanced relationship between cognitive reserve and depression in older adults, specifically examining how this association is moderated by the presence of animals in individuals’ lives. The inclusion of animals, whether as pets or service animals, introduces a unique dimension that has gained attention in mental health research, particularly due to the documented benefits of human–animal interaction on emotional well-being [29,30].

Thus, this research suggests the hypothesis that interaction with pets may enhance the protective effects of cognitive reserve, contributing to greater protection against depressive symptoms. Furthermore, we hypothesize that higher levels of cognitive reserve, operationalized as activities, serve as a more robust predictor for reducing Geriatric Depression Scale (GDS) scores among older adults. This hypothesis is grounded in the notion that sustained engagement in intellectually stimulating and socially enriching activities fosters cognitive flexibility, resilience, and emotional well-being, thereby mitigating the risk of depressive symptoms in later life.

The objective of this research was to analyze whether higher levels of cognitive reserve act as predictors for reducing depressive symptoms in older adults. Additionally, it aimed to investigate the potential moderating effect of pet companionship on the relationship between cognitive reserve and depression in older adults.

## 2. Methods

### 2.1. Participants

A total of 221 individuals participated in this study, recruited via convenience sampling through social media platforms such as Facebook, WhatsApp, and Instagram. The survey link was disseminated through posts and digital flyers shared in personal networks and in groups related to aging. The participants were divided into two groups: one composed of individuals living with pets and the other of individuals without pets.

The study included individuals all aged 59 or older, with a score of more than 14 points on the Telephone Interview for Cognitive Status Assessment—Modified Version (TICS-M) [31], who were literate, and who did not have uncorrected hearing problems that interfered with completing the instruments. There were 6 individuals who did not complete the entire questionnaire and were excluded from the study, resulting in a final sample of 215 participants.

### 2.2. Procedure and Ethics

The Research Ethics Committee of the Pontifical Catholic University of Rio Grande do Sul (PUCRS) approved this study under protocol number 65996522.5.0000.5336. All participants voluntarily agreed to take part in the study by signing an Informed Consent Form (ICF). Data was collected online via videoconferencing using the WhatsApp messaging app, and the information was stored on the Qualtrics platform. The interviews were conducted between late April and early September 2023.

### 2.3. Materials

Sociodemographic Questionnaire: The questionnaire was designed to collect data for the sociodemographic characterization of the sample, including variables such as age, gender, marital status, education level, and other relevant factors.

Cognitive Reserve Index Questionnaire (CRIq): The CRIq was used to assess the cognitive reserve of the participants. This questionnaire is composed of 20 items divided into three sections (CRI-Education, CRI-Working Activity, and CRI-Leisure Time). CRI-Education refers to the years of formal education and training courses, lasting at least six months, that the individual has completed throughout their life. CRI-Working Activity includes different work activities, divided into five levels according to the intellectual demands and personal responsibility required to perform the activity. For example, level 1 includes unskilled occupations such as gardener, domestic worker, driver, or call center operator. Level 2 includes manual or semi-skilled jobs such as cook, hairdresser, or nursing assistant. Level 3 includes skilled non-manual occupations such as musician, technician, preschool teacher, or real estate agent. Level 4 includes professional occupations such as lawyer, engineer, physician, or psychologist. Finally, level 5 includes highly professional occupations such as judge, university professor, researcher, or high-level executive. CRI-Leisure Time considers cognitively stimulating activities carried out by individuals during their free time, including intellectual, social, and physical activities. In its original version, this instrument’s Cronbach’s alpha coefficient is 0.73 [32]. This study used the version of the questionnaire translated into Brazilian Portuguese [33].

Telephone Interview for Cognitive Status—Modified Version (TICS-M): The TICS-M was employed to evaluate the cognitive functions of the participants. This tool is designed to provide a comprehensive assessment of various cognitive domains, such as memory, language, and attention, through a structured telephone interview [34]. In the modified version, two items were changed to include a measure that assessed delayed memory [35]. In this study, we applied the Brazilian version of the instrument, which has been translated and validated in a sample of elderly stroke survivors, with Cronbach’s alpha coefficient of 0.93. The Brazilian version of the questionnaire identified three domains: working memory, recent and delayed memory, and orientation [31].

Geriatric Depression Scale (GDS-15): The GDS is designed to measure symptoms of depression in the elderly. It assesses depressive symptoms experienced during the week preceding the administration of the questionnaire. Responses are classified as ‘yes’ or ‘no’, and scores range from 0 to 15, with scores below 5 indicating the absence of depressive symptoms and scores above 5 indicating their presence [36]. This study used the 15-item version, translated and validated for the Brazilian context, demonstrating adequate reliability, as shown by the Wilcoxon paired test (z = 1.60; *p* = 0.109), Spearman’s correlation (rho = 0.86; *p* < 0.001), and weighted Kappa (Kappa = 0.64) [37].

### 2.4. Design and Data Analysis

To examine the potential moderating effect of variable X on the relationship between variables Y and Z, a moderation analysis was conducted using the PROCESS macro for SPSS, version 23, developed by Hayes [38]. The analysis was performed with 10,000 bootstrapped samples to obtain robust estimates of the interaction effects.

## 3. Results

### 3.1. Participant Characteristics

The final sample consisted of 215 individuals, with a mean age of 69.13 years (SD = 6.89), ranging from 59 to 93 years. Among the participants, 74.4% were women, and 86.2% resided in the state of Rio Grande do Sul, Brazil. Regarding marital status, 51.8% were married or in a common-law marriage, and 37.2% had a higher education degree. In terms of pet companionship, 53% of participants had at least one pet, while 47% did not own any animals. These and other sociodemographic data are presented in Table 1.

### 3.2. Group Differences in Depression and Cognitive Reserve 

Based on the results of the independent samples *t*-tests under the Mann–Whitney U test, significant differences were observed in Geriatric Depression Scale (GDS) scores between the two groups described as individuals living with pets and individuals without pets (W = 6799.500, *p* = 0.020). A moderate positive association was found between group membership and GDS scores, with a rank-biserial correlation coefficient of 0.181 (95% CI: 0.028 to 0.326), indicating higher depressive symptomatology among individuals in one of the groups. In contrast, no statistically significant differences were detected between the groups in terms of education-related cognitive reserve. Descriptive statistics for depression and cognitive reserve scores across groups are presented in Table 2.

### 3.3. Correlation Analyses 

Spearman’s rho correlation analysis was conducted across the variables of interest (Table 3) and was further conditioned on the group variable (with and without animals; Table 4). The coefficients were similar in both cases.

### 3.4. Moderation Models: Total CRI Scores

The model for the whole CRI scores revealed a significant association between the predictors and the outcome (F(3, 211) = 4.24, *p* < 0.01). Both the group variable (Group: β = −0.2851, *p* < 0.05) and the CRI scores (β = −0.1535, *p* < 0.05) showed statistically significant coefficients, indicating their impact on GDS. With regard to the conditional effect of CRI on GDS at different values of the moderator, the relationship of group without animals was not statistically significant (β = −0.1082, *p* = 0.2916). Conversely, the group with animals had a significant negative effect on GDS (β = −0.1936, *p* < 0.05).

### 3.5. Bonferroni Correction for Multiple Testing

After controlling the family-wise error rate for the three principal hypotheses examined (Bonferroni-adjusted threshold α = 0.05/3 = 0.0167), the previously significant Mann–Whitney U difference in GDS score between participants with and without pets (uncorrected *p* = 0.020) no longer met the corrected criterion (*p* = 0.060). All effects that originally had *p* < 0.001 remained robust under this adjustment, while those reported at the conventional *p* < 0.05 level lost significance. Correlations and regression coefficients that had been significant at *p* < 0.01 continued to reach significance after correction because 0.01 < 0.0167. Thus, after accounting for multiple testing across the three main factors, only the strongest associations (i.e., those with *p* < 0.01, especially *p* < 0.001) persisted, indicating that the evidence for group differences in depressive symptoms was attenuated, whereas the most pronounced correlations remained reliable.

### 3.6. Moderation by Education-Related CRI

Secondly, when the model was examined through CRI scores related to education, the model was not statistically significant (*p* = 0.1631). Moreover, both the group and education-related CRI scores showed non-significant coefficients, indicating their limited influence on GDS (*p* = 0.3726). Not surprisingly, the conditional effect of X on Y at different values of the moderator was not statistically significant (*p* = 0.3348 versus *p* = 0.7859).

### 3.7. Moderation by Work-Related CRI

When work-related CRI scores were examined for the underlying model, the results approached the significance level: F(3, 211) = 2.4899, *p* = 0.0613. Once again, both the group and work-related CRI scores showed non-significant coefficients, indicating their limited influence on GDS (*p* = 0.7723). Not surprisingly, the conditional effect of X on Y at different values of the moderator was not statistically significant (*p* = 0.389 versus *p* = 0.193).

### 3.8. Moderation by Leisure Activity CRI

Lastly, the model was tested for activity-related CRI scores. The model demonstrated a statistically significant overall fit, as evidenced by an F-statistic of 6.7400 (*p* < 0.01). Activity-related CRI scores activity were statistically significant (*p* < 0.01), but group was not (*p* = 0.0837), for the GDS outcomes. The relationship of the group with animals was significant (*p* < 0.05), indicating a decrease in GDS scores. Conversely, for participants without animals, the effect remained statistically significant (*p* < 0.01), albeit with a slightly lower magnitude.

### 3.9. Moderation Models Across Groups

**Figure 1:**
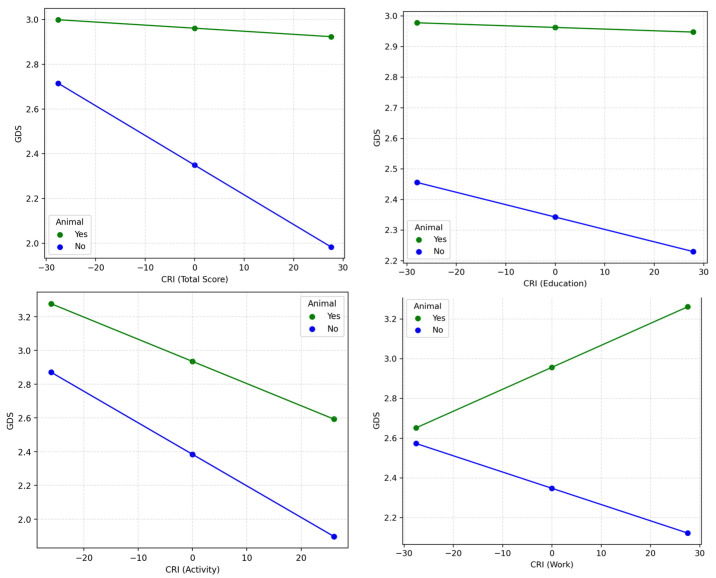
Moderation models across groups for relationship between CRI and GDS.

The results of the three models are depicted in terms of interaction between CRI scores and group on GDS in Figure 1. All estimates had a 95% confidence interval. Additionally, we mean-centered all the variables before analysis, and the standard errors were based on the HC3 estimator.

## 4. Discussion

This study provides evidence supporting the notion that higher levels of cognitive reserve (CR) are associated with lower GDS scores among older adults. This relationship underscores the importance of maintaining cognitive vitality through continued engagement in various activities, such as education, work, and leisure pursuits. The protective effect of cognitive reserve against depressive symptoms underscores the potential for interventions aimed at enhancing cognitive engagement to mitigate the burden of late-life depression.

Other studies also highlight the protective role of CR against depressive symptoms in older adults [3,4]. However, the research by Frau et al. found different results. In their study, although depression had a negative influence on executive function, moderated by levels of CR, the impact of depression was greater in individuals with higher levels of CR, suggesting that these individuals may be more susceptible to depression. The authors explain their findings by arguing that participants with low CR already exhibit reduced executive function, which would limit the impact of depression on these individuals’ cognitive abilities [39].

Furthermore, our results reveal that the relationship between CR and GDS score is moderated by the presence of animals in individuals’ lives. Specifically, older adults who have animals exhibit a stronger predictive effect of CR on reducing GDS scores compared to those without animals. This finding underscores the unique role of human–animal interaction in promoting emotional well-being and buffering against depressive symptoms in later life. This finding is consistent with the literature, which points to various psychological benefits associated with interaction with pets. According to Hui Gan et al., having a pet provides older adults with a sense of security, routine, and companionship, as well as a sense of purpose and meaning [40]. Regular interaction with animals helps reduce stress and anxiety levels, possibly due to the release of oxytocin [28]. Additionally, pets may contribute to the social functioning of older adults [41], and the presence of an animal also seems to motivate the maintenance of mobility and physical activity [42,43]. These factors can contribute to emotional stability and well-being, thus reducing depression in this population.

The loss of significance for the pet-ownership difference in GDS after Bonferroni adjustment suggests that the initially observed group effect was modest and susceptible to Type I inflation; once we protected against multiple testing, the evidence no longer supported a reliable association between pet ownership and depressive symptoms in this sample. In contrast, the correlations that survived correction, those originally below 0.01 and especially below 0.001, represent relationships strong enough to withstand a conservative error control and that therefore deserve greater theoretical weight. Collectively, these findings indicate that individual differences captured by the most robust correlations (e.g., specific facets of cognitive reserve) show consistent links to mood, whereas the simple presence or absence of a pet does not exert an independent effect once the broader analytical context is considered. Practically, this tempers claims that pet ownership per se mitigates late-life depression and redirects attention to the underlying cognitive and lifestyle variables that demonstrate more stable associations with mental health.

The study by Carr et al. supports this hypothesis, highlighting the role of pets in reducing depressive symptoms [21]. However, not all studies have found differences in depressive symptoms between older adults with and without pets [44,45]. Thus, we need to be cautious when interpreting these results, as it is necessary to consider the variability among individuals to better determine for whom or under which circumstances pet companionship may be beneficial [21].

Other studies highlight the protective role of pets against cognitive decline in old age. For example, the study by Friedmann et al. observed that older adults with pets showed better cognitive function [23]. Another study found a positive association between companionship with animals and having a better executive function [46]. The study by Shieu et al. showed that the relationship between having pets and cognitive function was stronger among those who had lived with animals for more than five years, suggesting that long-term companionship with animals may provide cognitive benefits for older adults [47].

The exact reason why pets may contribute to cognitive function is unknown; however, one possible explanation is that interacting with animals and the responsibility of caring for them may help individuals maintain high levels of activity, slowing cognitive decline [44]. Another hypothesis suggests that oxytocin may play a role in this association, as this hormone influences social cognition and memory encoding [28]. McDonough et al. argue that pets encourage their caregivers to continuously use specific cognitive processes, strengthening and enhancing these skills over time [46]. Moreover, pets may also provide their caregivers with social and physical stimulation, minimizing biological stress responses, which, in turn, may contribute to preserving cognitive ability or delaying cognitive dysfunction [44,48].

On the other hand, older adults with better cognitive function may be more likely to own pets [44]. However, the research by McDonough et al. does not support this hypothesis. The authors observed that companionship with multiple pets was associated with cortical thickness in older adults. Additionally, even after controlling variables such as cognition and brain health, specific brain effects verified in the study, such as increased volumes in the dorsal attention network and the limbic network, remained significant. These findings indicate that, regardless of cognitive status, pet companionship has a positive impact on cognitive function [46].

Considering studies suggesting that cognitive decline is related to increased depressive symptoms in older adults [49,50], we can deduce that maintaining cognitive function is essential for mental health in this age group. Thus, interaction with animals, by contributing to cognitive function, could also help reduce depressive symptoms.

Interestingly, not all subfactors of CR exert the same influence on GDS scores. While engagement in activities related to education and work did not significantly predict lower GDS scores, the presence of animals amplified the predictive power of CR, particularly in the context of leisure activities. This highlights the differential impact of various CR subfactors and underscores the importance of considering the multidimensional nature of cognitive engagement in promoting mental health in older adults.

The literature indicates that leisure activities are important in reducing depression [51]. Additionally, these activities seem to be associated with increased social interaction in older adults [52]. Considering the role of animals as social facilitators [53], engaging in leisure activities alongside pet companionship could enhance the protective effect of CR against depressive symptoms.

Moreover, leisure activities seem to be an important component of CR. According to the research by Farina et al., engaging in activities such as crossword puzzles, learning another language, and using electronic devices were associated with better cognitive function [54]. The authors concluded that these, among other factors, contribute to CR. Another study showed that participation in leisure activities is associated with protection against cognitive decline in older adults [55]. Therefore, unlike work- and education-related activities, which tend to demand greater cognitive effort, leisure activities are more flexible and may involve greater socialization, leading to increased well-being, which could explain these findings.

However, the relationship between CR and depression appears to be complex. Although some authors highlight that the relationship between cognition and depressive symptoms is unidirectional [9], a literature review investigating the variables that make up CR showed a negative correlation between CR and depression [56], indicating that there may be a bidirectional effect between the two variables.

Additionally, as mentioned earlier, pet caregivers appear to have better cognitive function, likely due to the increased stimulation generated by the responsibility of caring for animals [44,57]. Interaction with pets was also associated with larger brain structures, especially among dog caregivers [46]. The active model of CR is believed to be multidimensional, as various activities can benefit cognition, while numerous factors may indirectly influence CR [56]. Thus, there is a hypothesis that pets could also be considered an indirect component of CR.

The present research provides robust evidence of the moderating role of pets in the relationship between CR and GDS. However, some limitations should be considered when interpreting the results. First, the sample mainly consists of older adults with high education levels, elevated cognitive reserve, and good cognitive status. Additionally, recruitment was conducted via social media platforms, which may have introduced a selection bias, as older adults active on social media tend to have more education and better socioeconomic conditions than the general population. This discrepancy may limit the generalizability of the findings. Furthermore, important information about the relationship with the pet, such as the length of companionship and the species of the animal, was not included in the analyses, limiting the understanding of the specific and long-term impacts of this relationship. Finally, the data were obtained through questionnaires, which may introduce response bias.

Future studies should use a longitudinal design to assess pet companionship over time. It is also important to conduct studies with larger and more diverse samples in terms of demographic factors (socioeconomic and educational levels), which would help assess whether these effects can be observed in different profiles of older adults.

## 5. Conclusions

In conclusion, our study provides evidence that cognitive reserve, particularly in the form of engagement in activities, serves as a protective factor against late-life depression, particularly in individuals with animals. The presence of animals enhances the beneficial effects of cognitive reserve on mood, which is of interest for the therapeutic potential of human–animal interaction in promoting mental health in older adults. These findings underscore the importance of adopting a holistic approach to cognitive aging interventions that encompasses both cognitive engagement and social support mechanisms.

## Figures and Tables

**Table 1:** Demographic characteristics of participants.

Variables	Total n (%)	Pet Owners n (%)	Non-Pet Owners n (%)
Gender			
Women	160 (73.4)	88 (77.2)	72 (71.3)
Male	55 (25.2)	26 (22.8)	29 (28.7)
States of the Country			
Goiás (GO)	1 (0.4)	0 (0.0)	1 (0.9)
Minas Gerais (MG)	6 (2.7)	5 (4.4)	1 (0.9)
Paraná (PR)	3 (1.4)	2 (1.7)	1 (0.9)
Rio de Janeiro (RJ)	2 (0.9)	0 (0.0)	2 (1.9)
Rio Grande do Sul (RS)	188 (86.2)	99 (86.8)	89 (88.1)
Santa Catarina (SC)	7 (3.2)	6 (5.2)	1 (0.9)
São Paulo (SP)	8 (3.7)	2 (1.7)	6 (5.9)
Marital status			
Married/Common-Law Marriage	113 (51.8)	66 (57.9)	47 (46.5)
Divorced/Separated	39 (17.9)	16 (14.0)	23 (22.8)
Single	25 (11.5)	15 (13.1)	10 (9.9)
Widow(er)	38 (17.4)	17 (14.9)	21 (20.8)
Education Level			
Incomplete Primary Education (Up to 4 Years of Study)	8 (3.7)	3 (2.6)	5 (5.0)
Incomplete Secondary Education (Less than 11 Years of Study)	10 (4.7)	5 (4.4)	5 (5.0)
Complete Secondary Education (Up to 11 Years of Study)	38 (17.7)	21 (18.4)	17 (16.8)
Incomplete Higher Education	18 (8.4)	9 (7.9)	9 (8.9)
Complete Higher Education	80 (37.2)	41 (36.0)	39 (38.6)
Postgraduate (Specialization, Master’s, or Doctorate)	61 (28.4)	35 (30.7)	26 (25.7)
Employment Status			
Employed	15 (7.0)	9 (7.9)	6 (5.9)
Self-Employed	11 (5.1)	10 (8.8)	1 (1.0)
Retired	146 (67.9)	74 (64.9)	72 (71.3)
Retired but Still Working	40 (18.6)	20 (17.5)	20 (19.8)
Never Worked	3 (1.4)	1 (0.9)	2 (2.0)

**Table 2:** Descriptive statistics across groups.

	CRIq_Education_ Total		CRIq_Work_Total		CRIq_Leisure Activities_Total		CRIq_Total		GDS	
	No	Animal	No	Animal	No	Animal	No	Animal	No	Animal
Mean	130.059	126.658	116.772	116.711	106.030	110.605	123.277	123.816	3.000	2.333
Std. Deviation	21.830	19.279	22.370	20.971	19.185	21.673	22.168	19.430	2.470	2.115
Skewness	0.380	0.109	0.096	0.778	−0.066	0.687	−0.063	0.174	1.471	1.671
Kurtosis	−0.092	0.343	−0.186	1.287	0.022	0.886	−0.670	0.413	2.855	3.767
Minimum	85.000	71.000	69.000	74.000	59.000	66.000	72.000	79.000	0.000	0.000
Maximum	185.000	169.000	173.000	190.000	154.000	182.000	168.000	182.000	13.000	12.000

**Table 3:** Spearman’s correlations.

		Spearman’s Rho		Lower 95% CI	Upper 95% CI
CRIq_education_Total	CRIq_work_Total	0.479	***	0.368	0.576
CRIq_education_Total	CRIq_leisure activities_Total	0.294	***	0.167	0.412
CRIq_education_Total	CRIq_Total	0.764	***	0.702	0.814
CRIq_education_Total	GDS	−0.057		−0.190	0.077
CRIq_work_Total	CRIq_leisure activities_Total	0.309	***	0.183	0.426
CRIq_work_Total	CRIq_Total	0.772	***	0.711	0.821
CRIq_work_Total	GDS	0.001		−0.133	0.135
CRIq_leisureactivities_Total	CRIq_Total	0.687	***	0.610	0.752
CRIq_leisureactivities_Total	GDS	−0.278	***	−0.397	−0.149
CRIq_Total	GDS	−0.147	*	−0.275	−0.014

* *p* < 0.05, *** *p* < 0.001.

**Table 4:** Spearman’s correlations conditioned on group variable.

		Spearman’s Rho		Lower 95% CI	Upper 95% CI
CRIq_education_Total	CRIq_work_Total	0.479	***	0.368	0.576
CRIq_education_Total	CRIq_leisure activities_Total	0.294	***	0.167	0.412
CRIq_education_Total	CRIq_Total	0.764	***	0.702	0.814
CRIq_education_Total	GDS	−0.057		−0.190	0.077
CRIq_work_Total	CRIq_leisure activities_Total	0.309	***	0.183	0.426
CRIq_work_Total	CRIq_Total	0.772	***	0.711	0.821
CRIq_work_Total	GDS	0.001		−0.133	0.135
CRIq_leisureactivities_Total	CRIq_Total	0.687	***	0.610	0.752
CRIq_leisureactivities_Total	GDS	−0.278	***	−0.397	−0.149
CRIq_Total	GDS	−0.147	*	−0.275	−0.014

* *p* < 0.05, *** *p* < 0.001.

## Data Availability

Data are available on request from the first authors.
